# Computed Tomography-Guided Bronchoscopy With an Ultrathin Fiberscope

**DOI:** 10.1155/DTE.2.229

**Published:** 1996

**Authors:** Toshiaki Kobayashi, Kayako Shimamura, Kohzoh Hanai, Masahiro Kaneko

**Affiliations:** 1 Department of Endoscopy National Cancer Center Hospital 1-1, Tsukiji 5-chome Chuo-ku Tokyo 104 Japan; 2 Department of Cytology National Cancer Center Hospital Tokyo Japan; 3 Department of Diagnostic Radiology National Cancer Center Hospital Tokyo Japan

## Abstract

Bronchoscopy was performed under computed tomography (CT) guidance using an ultrathin fiberscope in a patient with a fluoroscopically invisible lesion that was visualized by CT in the right S^8^ and with poor pulmonary function. Under local anesthesia, the ultrathin fiberscope (3 mm in diameter) was inserted close to the lesion (1.5 mm in diameter) under direct visual guidance, and a brush was inserted into the lesion under CT guidance. Cytologic specimens obtained by the brush and washing revealed adenocarcinoma. This is the first report of CT-guided bronchoscopy, which is a new examination method for peripheral small lung lesions and is a less invasive examination than either endoscopic examination with a conventional bronchoscope or open lung biopsy, especially for those with poor pulmonary function.


*Diagnostic and Therapeutic Endoscopy*, 1996, Vol. 2, pp. 229–232
Reprints available directly from the publisher
Photocopying permitted by license only

© 1996 OPA (Overseas Publishers Association)
Amsterdam B. V. Published in The Netherlands
by Harwood Academic Publishers GmbH
Printed in Singapore

### Computed Tomography-Guided Bronchoscopy with an Ultrathin Fiberscope

TOSHIAKI KOBAYASHI, KAYAKO SHIMAMURA, KOHZOH HANAI, and MASAHIRO KANEKO

*Departments of Endoscopy (T.K., M.K.), Cytology (K.S.), and Diagnostic Radiology (K.H.), National Cancer Center Hospital, Tokyo, Japan*

*(Received November 3, 1995; in final form December 25, 1995)*

Bronchoscopy was performed under computed tomography (CT) guidance using an ultrathin fiberscope in a patient with a fluoroscopically invisible lesion that was visualized by CT in the right $S^8$ and with poor pulmonary function. Under local anesthesia, the ultrathin fiberscope (3 mm in diameter) was inserted close to the lesion (1.5 mm in diameter) under direct visual guidance, and a brush was inserted into the lesion under CT guidance. Cytologic specimens obtained by the brush and washing revealed adenocarcinoma. This is the first report of CT-guided bronchoscopy, which is a new examination method for peripheral small lung lesions and is a less invasive examination than either endoscopic examination with a conventional bronchoscope or open lung biopsy, especially for those with poor pulmonary function.

**KEY WORDS:** Bronchoscopy, CT, lung cancer, CT-guided bronchoscopy, biopsy, cytology

### INTRODUCTION

Percutaneous needle biopsy under fluoroscopic guidance and bronchoscopy are standard examinations for lesions in the peripheral lung. When lesions are small, but visible fluoroscopically, percutaneous needle cytology is preferred to bronchoscopic examinations. Patients with fluoroscopically invisible lesions were either subjected to open lung biopsy or followed-up, in which case there was the risk of progressive disease. Recently, computed tomography (CT)-guided needle biopsy has been used for lesions that are invisible fluoroscopically. In addition, video-assisted thoracoscopic biopsy is also indicated for lesions that are strongly suspected of being malignant. In video-assisted thoracoscopic biopsy, even smaller lesions can be resected by applying new techniques such as needle implantation and a highly sensitive sensor (1,2). However, thoracoscopic biopsy is invasive, and even CT-guided needle biopsy is dangerous for patients with poor pulmonary function because of the possibility of pneumothorax. Concerning bronchoscopy, the only attempts to approach tiny lesions have been the individual efforts of bronchoscopists to improve their own experience and technique. We adapted the CT-guided needle biopsy technique to bronchoscopy. CT-guided bronchoscopic brushing cytology performed using an ultrathin fiberscope on a patient with a fluoroscopically invisible lesion and with poor pulmonary function yielded a cytologic diagnosis of adenocarcinoma.

### CASE REPORT

A 69-year-old man in whom an infiltrative shadow was detected in left $S^{1+2}$ on chest x-ray films during follow-up for chronic obstructive pulmonary disease (COPD) was referred to our hospital. Bronchoscopy performed at the previous hospital showed no malignancy. Subsequent CT performed at our hospital revealed another lesion in the right $S^8$, the CT findings of which suggested malignancy. Bronchoscopic curettage of both lesions showed no malignancy. Transbronchial lung biopsy (TBLB) of the left lesion showed no malignancy. The right lesion was not examined at that time because the lesion was invisible fluoroscopically. CT-guided needle biopsy was con-

Address for correspondence: Toshiaki Kobayashi, M.D., Ph.D., Department of Endoscopy, National Cancer Center Hospital, 1-1, Tsukiji 5-chome, Chuo-ku, Tokyo 104, Japan. Tel: 81-3-3542-2511; Fax: 81-3-3542-3815.

sidered, but the procedure was considered to be contraindicated because of the possibility of severe pneumothorax combined with poor pulmonary function due to COPD; VC was 2.83 liters and $FEV_{1.0}$ was 1.2 liters. Therefore, CT-guided bronchoscopy was planned after obtaining informed consent.

Under 4% lidocaine local anesthesia, the Pentax FUR-9P with a 3-mm distal rigid tip diameter and a 1.2-mm working channel was inserted orally without any premedication. The tip of the fiberscope was inserted into the right B8ai, which was confirmed as the target area by thin-section CT before the procedure, under direct visual guidance. The location of the tip of the fiberscope was confirmed by a CT system (Toshiba Xpress/SX) that allows real-time dynamic visualization in CT fluoroscopy mode (0.67 sec delay), then a thin brush (Pentax BC1610) was inserted toward the lesion under CT guidance. The tip of the brush and the tip of the fiberscope were clearly visualized on CT. After the location of the tip of the brush was confirmed to be exactly in the lesion (Fig. 1), cytologic specimens were obtained. Immediately after the brush was extracted, the tip of the fiberscope was wedged into the bronchus for hemostasis for 2 min. No hemorrhage was observed. Washing cytology was performed with 3 ml of physiologic saline. The patient reported less discomfort than on a previous conventional bronchoscopy. The amounts of brushing and washing cytologic specimens were sufficient for a cytologic diagnosis of adenocarcinoma.

### DISCUSSION

Bronchoscopy allows direct visualization of the central tracheobronchial tree and is considered to be the best examination to detect early-stage lung cancer. In addition, several new methods such as photodynamic diagnosis (3) and the lung imaging fluorescence endoscopic device system (4) have been attempted to detect minute lesions.

In cases of peripheral lesions, bronchoscopic examinations such as TBLB TV (television) are effective approaches to obtain definitive diagnosis after their detection. Percutaneous needle biopsy can contribute, especially in patients with small lesions. CT-guided needle biopsy is an effective examination for fluoroscopically invisible lesions (5). Video-assisted thoracoscopic biopsy can be performed for lesions that are difficult to diagnose with standard techniques without having to subject the patients to open lung biopsy. Although definitive diagnosis of peripheral pulmonary lesions is possible, thoracoscopic biopsy is still an invasive procedure for patients. Even percutaneous needle biopsy is an invasive procedure involving the possibility of pneumothorax and he-

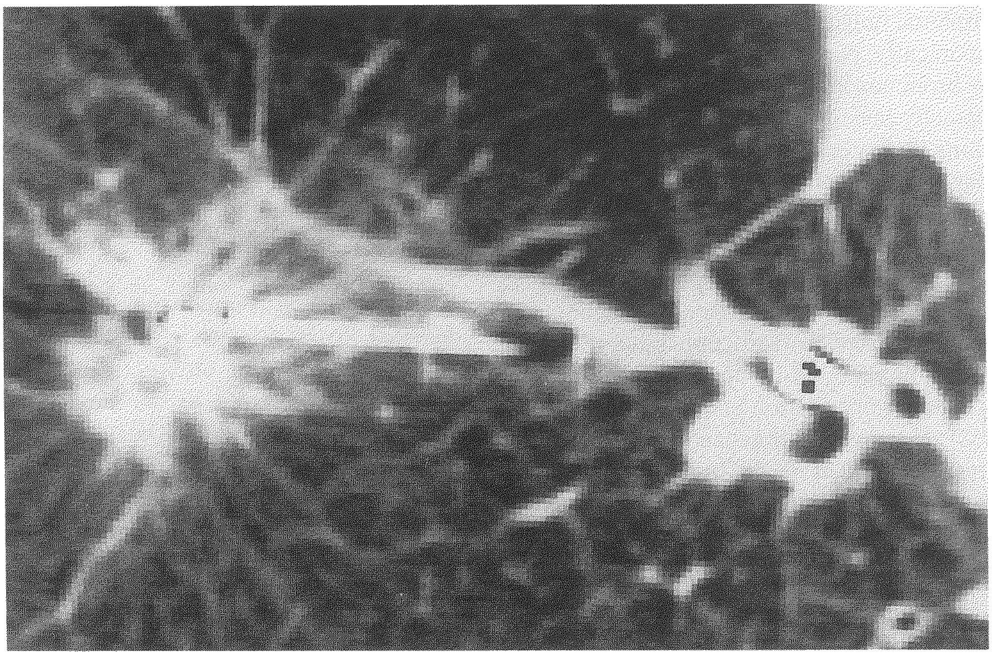

**Figure 1** The tip of the brush and the tip of the fiberscope were visualized clearly on CT. The tip of the brush was considered to be exactly in the lesion.

morrhage, which are particularly worrisome in patients with poor pulmonary function.

Percutaneous needle biopsy was contraindicated in the present patient because of the high possibility of pneumothorax and deterioration in condition associated with his poor pulmonary function caused by COPD. No problems had developed on three preceding bronchoscopies, and this allowed us to consider another bronchoscopy. The problem was the fact that the left lesion was invisible fluoroscopically and even on lateral chest x-ray film. In general, when a malignant specimen is obtained, it is definitively diagnostic; however, a benign specimen is not, because it is possible that the target lesion was not accurately biopsied. In this respect, CT is one of the best ways to ensure the accuracy of targeting. Considering these conditions, the best possible procedure was considered to be a combination of bronchoscopy and CT, i.e., CT-guided bronchoscopy.

The fiberscope used was the Pentax FUR-9P, originally designed for the urinary tract. It was considered more appropriate for this procedure than standard bronchofiberscopes because of several characteristics: 1) a thinner distal rigid portion diameter (3.0 mm); 2) a relatively wider working channel (1.2 mm); 3) a longer insertion portion (700 mm), which allows the insertion to more peripheral bronchi and gives extra length to cover the distance from the patient around the CT system; 4) a more rigid insertion portion, which enables easier insertion into deeper and thinner bronchi; and 5) a longer light guide, allowing connection to be made around the CT system. The CT system (Toshiba Xpress/SX) allows real-time (0.67 sec delay) dynamic image visualization in CT fluoroscopy mode, which was considered optimal to facilitate the procedure.

Our experience with CT-guided bronchoscopy suggested the following.

1. Although CT-guided bronchoscopy required a longer examination time than conventional bronchoscopy, the procedure time will be shortened with more experience and better preparation.

2. Regular local anesthesia with 4% lidocaine without any premedication allowed the patient to tolerate the procedure better than three previous bronchoscopies. Almost no cough was observed during the procedure despite the prolonged examination time. This was considered to be related to the use of an ultrathin fiberscope.

3. The ultrathin fiberscope could be inserted into peripheral bronchi close to the lesion under direct visualization. However, some difficulty on insertion was experienced because the insertion portion of the fiberscope still tended to bend, and the tip was slightly thick. Therefore, more rigid and thinner bronchofiberscopes are required for CT-guided bronchoscopy. The length of the insertion portion was appropriate for the procedure. The light guide should be longer because of the large size of the CT system, which requires the light source to be placed at a distance from the patient. Small portable light sources are also needed because CT rooms are not designed for bronchoscopy at present.

4. The tip of the brush, the tip of the fiberscope, and the insertion portion were visualized clearly on CT. This means the origin of specimens can be defined absolutely, and the possibility of malignancy can be almost definitely denied in cases of benign specimens. In addition, access to tiny lesions such as very early stage lung cancers visualized only on CT and tiny pulmonary metastases will become possible. Even conventional forceps passed through a conventional bronchofiberscope can be guided by CT to fluoroscopically invisible lesions for CT-guided TBLB.

5. Visualization on CT of structures at approximately 90 degrees to the CT section was easier than parallel structures. This means that certain locations have easier access, for example, lesions in $S^1$ are easier to reach than those in $S^3$.

6. Confirmation of the tip of the fiberscope was slightly difficult because of respiratory movement. Therefore, the fiberscope should be inserted as far as possible to peripheral bronchi under direct visual guidance. For successful results, the location of the lesion should be confirmed by thin-section CT before the procedure.

7. Real-time dynamic image visualization in the CT fluoroscopy mode is not always better than conventional CT visualization because the speed of the CT system is unable to compensate for movement caused by breathing. In this situation, we have three moving factors: breathing, CT, and examination instruments. For easy detection, we recommend stabilizing the breathing and examination tools to confirm the location of examination tools after inserting them into a target bronchus.

8. No hemorrhage was observed after obtaining cytologic specimens. Harvesting of cytologic specimens by a thin brush or washing is the least invasive approach. Even if hemorrhage were to occur, if the tip of the fiberscope is inserted into a narrow bronchus, the hemorrhage should be controlled easily.

9. Cytologic specimens obtained by a thin brush and washing with 3 ml of physiologic saline were sufficient for a cytologic diagnosis. If washing cytologic specimens are sufficient for diagnosis, CT-guided bronchoscopy could be a minimally invasive diagnostic examination for peripheral lung lesions.

10. CT rooms are not designed for bronchoscopy. There was insufficient space for the examiner around the CT.

Therefore, we had to prepare a fiberscope with a long insertion portion and a long light guide. In the future, CT rooms should be designed to allow endoscopy to be performed.

More studies will be required to assess the value of CT-guided bronchoscopy itself. However, we consider through this first experience that more accurate, less invasive, and less uncomfortable examinations for peripheral small lung lesions can be performed by CT-guided bronchoscopy using ultrathin fiberscopes. In patients with small peripheral lung lesions, especially with lesions not recognizable on normal x-ray films but visible on CT and/or with poor pulmonary function, CT-guided bronchoscopy is a new weapon in the diagnostic arsenal.

### REFERENCES

1. Plunkett MB, Peterson MS, Landreneau RJ, et al. Peripheral pulmonary nodules: preoperative percutaneous needle localization with CT guidance. Radiology 1992;185:274–276.
2. Ohtsuka T, Furuse A, Kohno T, et al. Application of a new tactile sensor to thoracoscopic surgery: experimental and clinical study. Ann Thorac Surg 1995;60:610–614.
3. Kato H, et al. Photodynamic diagnosis in respiratory tract malignancy using an excimer dye laser system. J Photochem Photobiol 1990;6:189–196.
4. Lam S, MacAulay C, Leriche JC, et al. Early localization of bronchogenic carcinoma. Diagn Ther Endosc 1994;1:75–78.
5. vanSonnenberg E, Lin AS, Deutsch AL, et al. Percutaneous biopsy of difficult mediastinal, hilar, and pulmonary lesions by computed tomographic guidance and a modified coaxial technique. Radiology 1983;148:300–302.

